# Attachment Style, Early Childhood Trauma, Alexithymia, and Dissociation Among Persons Addicted to Alcohol: Structural Equation Model of Dependencies

**DOI:** 10.3389/fpsyg.2019.02957

**Published:** 2020-01-24

**Authors:** Elżbieta Zdankiewicz-Ścigała, Dawid Konrad Ścigała

**Affiliations:** ^1^Faculty of Psychology, SWPS University of Social Sciences and Humanities, Warsaw, Poland; ^2^Institute of Psychology, Faculty of Applied Social Sciences, The Maria Grzegorzewska University, Warsaw, Poland

**Keywords:** attachment style, trauma, alexithymia, dissociation, addiction

## Abstract

**Aim:**

Attachment theory is a broadly used paradigm for understanding human affective development. It is recognized that alexithymia is a key factor responsible for the non-adaptive strategies of regulating emotions in people addicted to alcohol. In addition, an important role is attributed to early childhood trauma and dissociation. The theoretical model was examined, in which connections between attachment styles, trauma, and alexithymia and dissociation were investigated in persons addicted to alcohol.

**Methods:**

The total number of study participants amounted to 268 persons, including 116 women (43% of all subjects) and 152 men (57% of all subjects) at the age of 18–73 (*M* = 39.19; *SD* = 13.45). In order to measure the analyzed variables, the following questionnaires were applied: Michigan alcoholism screening test (MAST), attachment styles questionnaire (ASQ), 20-item Toronto Alexithymia Scale (TAS-20), traumatic experiences checklist (TEC), and curious experiences survey (CES).

**Results:**

A comparative analysis between the group of alcohol addicts and non-addicts showed statistically significant differences related to attachment style, intensity of trauma, alexithymia, and dissociation. With structural equation models (i.e., AMOS and GLS), the adjustment of theoretical model to data was examined, which allowed the description of dependency paths. As a result of the conducted analysis of paths, it was found out that the model was accurately fitted to data, but only when an impact path related to a direct connection between an attachment style and an addiction was deleted. This impact is indirect, and from one side, it results from affective and cognitive deficits, i.e., alexithymia, and on the other side, from the intensity of traumatic experiences. No direct impact of dissociation on the development of an inclination to addiction was found, if contextual variables, i.e., alexithymia and trauma, are taken into account. The strongest direct relation was proven in the case of the anxious-ambivalent attachment style and alexithymia (β = 0.389; *p* < 0.01) and avoidant attachment style and alexithymia (β = 0.497; *p* < 0.01), which turned out a strong predictor fostering the development of alexithymia and the occurrence of traumas related to emotional negligence and mental violence and finally addiction.

**Conclusion:**

Our studies revealed how important it is to investigate the role of individual variables in the context of developmental models. An extremely important element of the scientific achievement presented here is showing pillars of trauma, alexithymia, and dissociation in their cumulative impact on the development of emotional disorders resulting in addiction.

## Introduction

The definition of alcohol dependence as a set of behavioral and physical symptoms in people who consume large amounts of alcohol, including alcohol withdrawal syndrome, tolerance, and craving, has not changed in the *diagnostic and statistical manual of mental disorders* (DSM)-5 classification (APA, 2013). People with such disorders often lack awareness of the emotions experienced. This, in turn, implies deficits in the cognitive processing of emotional arousal and, finally, disorders in the regulation and self-regulation of emotions (Taylor et al., 1997). Deficits in recognizing and identifying specific emotions experienced by the individual disturb the selection of adequate and effective strategies for their regulation. As a result, strategies will be largely random or based on short-term consequences and therefore inadequate (Sim and Zeman, 2004). Accordingly, in this view, addicts have a high level of alexithymia (Thorberg et al., 2009). From 45% to 67%, alcohol-dependent individuals are estimated to show a high level of alexithymia (Thorberg et al., 2011). Alexithymia is considered as a personality trait with a dimensional nature (Taylor et al., 1991; Messina et al., 2014; Poquérusse et al., 2018). Characteristics of alexithymia include (1) difficulty identifying feelings and distinguishing between feelings and bodily sensations of emotional arousal, (2) difficulty describing feelings toward other people, (3) externally oriented cognitive style, and (4) low perspective taking, as well as difficulty describing and understanding the emotions of others (Saymur et al., 2013). The individuals with a high level of alexithymia may attempt to regulate their emotional states behaviorally rather than cognitively. Krystal and Raskin suggest that the substance-dependent individuals felt their affections globally and somatically and had considerable difficulty tolerating painful affects (Krystal, 1988).

Early childhood experiences, similarly to building foundations, determine how stable, and solid construction may be built upon them. Although they do not determine a specific course of adaptive functioning, they restrict its form in a probabilistic manner, by increasing or decreasing the probability of certain phenomena’s occurrence (Bowlby, 1988; Sroufe, 2005; Thompson, 2008). The essence of development processes is the fact that both the development of competences and their absence, i.e., inadaptation, result from the same transaction and cumulative processes, which take place in the course of individual development (Yates et al., 2011). A number of researchers, who investigate alcohol addictions (e.g., Martinotti et al., 2008, 2009; Cierpiałkowska, 2010; Ryszkowski et al., 2015; Lyvers et al., 2018a), emphasize that alcohol addictions result from an individual’s disorder, i.e., emotional immaturity, and alcohol may constitute the means to handle difficult situations. As appears from the above, essential for dependence/addiction are mechanisms responsible for the release of craving or the modulation of desire and craving in such a manner that the process is regulated by other mental processes or blocked by those processes (Martinotti et al., 2008; Thorberg et al., 2011). The substance causes the change of emotions toward the positive, giving the opportunity to divert attention from problems, boredom, overwhelming, and stagnation/apathy. Often difficult to capture for a drinking individual is a moment of transition from occasional and relatively controlled consumption of alcohol to addiction. Researchers agree that one of the key elements that cause dependent individuals to refer to psychoactive substances is the state characterized by an increased desire or willingness to use those substances, e.g., to drink alcohol, to feel its “beneficial” effects, or to compulsively search for it (Addolorato et al., 2005; Pocuca et al., 2018). Moreover, as was proven by Dragan (2016), the regulation of negative emotional states is connected with positive metacognitive beliefs, which problematically or hazardously drinking individuals have regarding alcohol.

There is an extensive database of study results in literature which connect attachment styles and alexithymia (Hexel, 2003; Montebarocci et al., 2004; Bekker et al., 2007); alexithymia and addiction (Taylor et al., 1990; Morie and Ridout, 2018; Thorberg et al., 2009, 2011); attachment style and alexithymia in substance use disorders (De Rick and Vanheule, 2006, 2007; Hiebler-Ragger and Unterrainer, 2019; Lyvers et al., 2019); trauma and alexithymia in substance-dependent inpatients (Evren et al., 2009); and finally, trauma, dissociation, and alexithymia with addiction (Craparo et al., 2014; Zdankiewicz-Ścigała and Ścigała, 2018a, b; Costanzo et al., 2020). Individuals who experience affective overload chronically may become used to expressing their affect (van der Kolk and McFarlane, 1996) by developing a compensative, non-verbal strategy, such as pathological drinking to break the feeling of mental numbness or avoid and manage intensive, seemingly uncontrolled emotions in order to understand and express those emotional states. At the time, whenever this happens, the expression of emotions typically takes the oversized or even irrelevant or distorted form, reversely contributing to mental overwhelming with affect that is inconceivable for the experiencing individual (Yates, 2009) and repeating the regulation cycle of this affect with stimulants. Substance, i.e., alcohol, which was supposed to help, although it works temporarily, does not allow, after getting sober, dealing with emotions, and as a result, it becomes a generator of craving and obsessive thinking about the pleasure and relief which its consumption may bring. Mental integrity ensures relevant and coherent functioning both in the field of relations with the external world and within an individual’s internal world. Traumatic experiences, due to unexpected confrontation with death, risk to life, or threat to physical and mental integrity – at least temporarily – will evoke chaos in all fields of life, i.e., disintegration. Alexithymia and dissociation may overlap in the process of handling stressful situations, contributing, at the same time, to the increase of deficits in the affective and cognitive sphere of behavior regulation. An inclination to detachment (derealization and depersonalization) and emotional blindness have a common source, i.e., insecure attachment styles and early childhood traumas (Liotti and Farina, 2016; Zdankiewicz-Ścigała, 2017). There is an extensive database of study results in literature, which separately connects attachment styles, trauma, dissociation, and alexithymia with a tendency toward addiction. An inability to identify or name emotions (alexithymia), combined with an affective overwhelming condition (dissociation, i.e., disconnection), is frequently typical for persons who experience traumas. The study carried out by Craparo et al. (2014) includes the analysis of trauma, as well as dissociation and alexithymia, within the meaning of alcohol dependence origins. And the study carried out by Zdankiewicz-Ścigała and Ścigała (2018a) includes the analysis of trauma, temperament, and dissociation and alexithymia within the meaning of alcohol dependence origins. The main, negative consequence of anxious attachment styles and early childhood trauma is general emotional dysregulation, also visible on the biological level. It results from the disturbance of dynamic psycho-neuro-immunological balance of systems which take part in response to stress, due to the sensitization process (Schore, 2009a, b). This, in turn, is connected with susceptibility to emotional disorders. The available knowledge on neuronal correlates underlying alexithymia and dissociation enables more extensive explanation of obtained results (Craig, 2009; Sutherland et al., 2013; van der Velde et al., 2013; Morie et al., 2016). Alexithymia and dissociation, analyzed together as pillars of traumatic development, cause disintegration resulting in emotional dysregulation. The chaos is reinforced by strong fear, which may turn into general anxiety or adopt the form of other difficult-to-regulate and difficult-to-understand emotions (feelings of guilt, embarrassment, and anger), or non-specific affective states. Alcohol dependence, according to such understanding, may be perceived as a defensive response to stress and trauma. In addition, excessive alcohol consumption, or intoxication with other psychoactive substances, may serve for generating altered states of consciousness (chemical dissociation), preventing an individual from becoming aware of mental suffering (Craparo et al., 2014). In relation to the above, the abuse of alcohol may constitute an element comprising the set of psychopathological responses to trauma (De Bellis, 2002; Foa and Williams, 2010).

**FIGURE 1:**
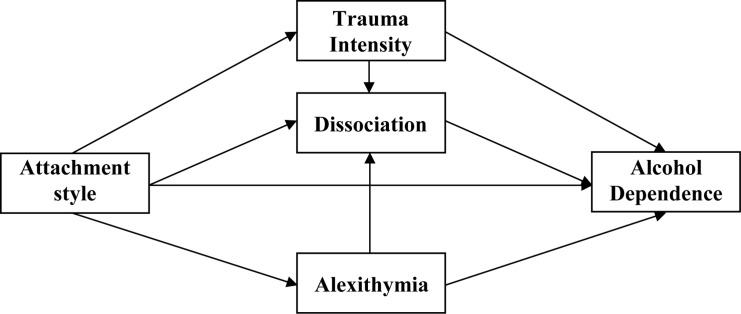
A graphical model presenting the nature of dependencies between attachment styles and trauma, dissociation, and alexithymia, in explaining an inclination to alcohol addiction risk. Source: Zdankiewicz-Ścigała (2017).

No theoretical *explicit* model or studies were presented so far, which would take account of all significant, content-related variables. Based on that, in the verified model, it was assumed that both alexithymia and dissociation contribute to the development of psychopathology in childhood and to the maintenance of non-adaptive mechanisms of coping with stress in adulthood. The selection of alcohol-dependent persons to verify the model of dependences was determined by the fact that in addicted persons, in a series of examinations, the high volume of traumatic experiences’ occurrence in childhood was proven, and as a result, the disorders of emotions’ regulation and self-regulation. The innovative nature or the new view, which we present, relates to a holistic approach toward describing aspects of traumatic development and explaining psychological mechanisms responsible for the development and maintenance of affective dysfunctions. Our intention was to identify a specific path of the etiological development of addiction from a psychological perspective. The heuristic character of the analyses is based on answering key questions about the pedigree of alexithymia and dissociation in the context of attachment styles and trauma and on responses to the role of attachment styles and trauma in the influence of alexithymia and dissociation on addiction. Due to partial data including several variables important for understanding the mechanism of the development and maintenance of alcohol dependence, authors have developed a theoretical model whose goal was to take into account all relevant factors. According to this, a direct impact of attachment styles on alcohol addiction was assumed. The following two analyzed paths relate to the relationship between attachment styles and addiction through alexithymia. The more anxious the attachment styles are, the stronger the impact on the level of alexithymia, and this in turn is more strongly related to addiction. The subsequent path in the model refers to the relationship between attachment styles and the intensity of traumas and dissociation. Trauma is perceived as an indirect factor that strengthens the direct impact of the attachment style on addiction. According to the data from other studies quoted above, it is recognized that alexithymia and trauma affect the inclination to pathological dissociation, and these mutual relationships increase a tendency to addiction. A direct impact of attachment styles and an indirect impact of trauma, alexithymia, and dissociation upon addiction are anticipated. The model has been illustrated in Figure 1.

## Materials and Methods

This study was carried out in accordance with the recommendations of the SWPS University of Social Sciences and Humanities Ethics Committee with written informed consent from all participants. All procedures performed in studies involving human participants were in accordance with the ethical standards of the institutional and/or national research committee and with the 1964 Helsinki declaration and its further amendments or comparable ethical standards. An ethics approval for this research was not required as per the SWPS University of Social Sciences and Humanities Ethics Committee’s guidelines and national regulations. Standardized questionnaires, which are used in psychological worldwide research, were exclusively used in the research procedure. This type of research is based on guidelines and procedures in accordance with applicable law and ethics but do not require individual consent. Consent to the study was approved by the appropriate authorities of the therapeutic departments and the patients themselves. It was carried out in addiction therapy departments by psychologists working permanently with patients. Before starting to fill in the questionnaires, they were asked to sign an informed consent form which specified all their tasks and rights. The total number of study participants amounted to 268 persons, including 116 women (43% of all subjects) and 152 men (57% of all subjects) at the age of 18–73 (*M* = 39.19; *SD* = 13.45). Based on a MAST questionnaire for diagnosing alcohol addiction, subjects were divided into three groups (a score below 4 points—a control group, i.e., without any apparent problem; 4 points—likely dependent/addicted; and over 5 points—alcoholics). Scoring 5 points or more indicated that a subject fulfilled the criteria of alcoholism according to DSM-IV-TR diagnostic criteria. The control group comprised 90 subjects, including 53 women and 37 men at the age of 21–64 (*M* = 35.12; *SD* = 1.40). The group of likely addicted comprised 14 subjects. Five of them were women and nine men at the age of 20–59 (*M* = 29.71; *SD* = 2.97). A group of alcohol addicted comprised 163 subjects: 57 women and 106 men at the age of 18–73 (*M* = 42.36; *SD* = 1). The study was carried out among patients of six addiction treatment centers in Warsaw from the 8-week abstinence-based inpatient treatment program combining intensive group and individual therapy, as well as elements of 12-step facilitation and relapse prevention. The study was conducted at the end of the detoxification processes of alcohol-dependent inpatients. The study was carried out from October 2014 to January 2015.

### Methods

A questionnaire, which was used in the study, included questions related to age, sex, marital status, education, and information on whether, and if yes, how long, for reasons related to alcohol drinking, a given person had participated in the therapy.

### Measures

#### Attachment Style

The attachment styles questionnaire (ASQ) designed by Plopa (2008) based on the Hazan and Shaver (1987) theory assumes the existence of three attachment styles. The questionnaire’s purpose is to measure an independent variable, i.e., attachment style, in terms of its three measurements: secure, ambivalent, and avoidant. ASQ is used to measure the style of attachment to his/her partner in the current marriage. Responses are given with the use of a 7-point Likert grading scale, and they range from (1) totally disagree to (7) totally agree with the statement. Each scale contains eight statements (all scales include 24 statements), so the raw results are within 8–56 points. In the Cronbach alpha reliability test, particular scores were as follows: secure style α = 0.91, ambivalent style α = 0.78, and avoidant style α = 0.80. In the study, we can obtain a constellation of different styles, so each of the subjects is described using three indicators that determine the intensity of the three attachment styles (Plopa, 2008).

#### Addiction to Alcohol

The Michigan alcoholism screening test (MAST) (Selzer, 1971) was applied to investigate the level of alcohol addiction. The questionnaire is a screening test; it comprises 24 questions to which a subject responds “yes” or “no.” Questions are valued 0 to 5 points. The score on a general scale is from 0 to 53 points. Obtaining 5 or more points means the statement of alcoholism, according to DSM-IV-TR classification. MAST provides results of sufficient reliability for research purposes, but considerable caution is advised when applying the one (Shields et al., 2007). As studies show, results of MAST are less reliable in the case of women and non-clinical cases; however, applying this tool in a clinical group, as a quantitative indicator of alcohol addiction, is fully justified. Shields (2003) has validated methods for alcohol screening measures relative to current standards. Results suggest that the alcohol use disorders identification test (AUDIT), MAST, and Short Michigan Alcoholism Screening Test (SMAST) are generally capable of producing scores of sufficient reliability for most basic research purposes, and individuals administering these measures can do so with confidence in such situations. The Polish version of the MAST test is described as a tool with satisfactory psychometric properties (Cierpiałkowska, 2001). The MAST results have been compared with those from other popular screening tools which facilitate making the diagnosis of addiction, e.g., AUDIT. These comparisons have shown that the results obtained from both tools are characterized by a high level of convergence (Woronowicz, 2009). In this study, Cronbach’s α coefficient on the level of 0.917 means a good reliability of the scale.

#### Trauma

The traumatic experiences checklist (TEC) (Nijenhuis et al., 2002) was applied to investigate the intensity of traumatic experiences. In the study, a general scale of traumatic experiences was used exclusively, but the questionnaire also enables the calculation of scores for particular trauma categories. A participant responds to 29 questions which refer to potentially traumatic events. For each question, individuals are required to provide information on whether a given event took place in their lives; their age when the trauma was suffered; and the duration and the subjective level of the trauma’s impact on their lives (choice within the range of 1 to 5). The more points there are, the greater the intensity of traumatic experiences. Cronbach’s α coefficient on the level of 0.91 means a good reliability of the scale, and studies which compare it to the Stressful Life Experiences Questionnaire (SLESQ) prove its high accuracy (*r* = 0.77; *p* < 0.0001) (Nijenhuis et al., 2002).

#### Alexithymia

The 20-item Toronto Alexithymia Scale (TAS-20) (Parker et al., 1993) was applied to investigate the level of alexithymia. Apart from the general level of alexithymia, the questionnaire allows for estimating separate scales for dimensions such as “difficulties in describing feelings” (DDF); “difficulties in identifying feelings” (DIF); and “externally oriented thinking” (EOT). The questionnaire comprises 20 test items. Each item has a five-degree Likert scale (1, strongly disagree; 2, partially disagree; 3, no opinion; 4, partially agree; and 5, strongly agree). The scale is from 20 to 100 points. It is a reliable and accurate tool. In relation to the Polish version, Cronbach’s α coefficient is 0.73 for the general score; 0.55 for the “difficulties in verbalizing feelings” scale; 0.71 for the DIF scale; and 0.51 for the “operational style of thinking” scale. In relation to the Polish version, Cronbach’s α coefficient is 0.73 for the general score; 0.55 for the “difficulties in verbalizing feelings” scale; 0.71 for the DIF scale; and 0.51 for the EOT scale (Ścigała et al., 2020).

#### Dissociation

The curious experiences survey (CES) (Goldberg, 1999) was applied to investigate a tendency toward dissociation. The survey enables estimating the general level of tendency toward dissociation, and the scales included “amnesia,” “absorption,” and “depersonalization.” The survey comprises 31 test items. Each item has a five-degree Likert scale (1, it never happens to me; 2, it rarely happens to me; 3, it sometimes happens to me; 4, it often happens to me; and 5, it always happens to me). The scale is from 31 to 155 points. Psychometric tests carried out for the original language version indicate that it is a reliable tool; Cronbach’s α coefficient for calculating reliability is 0.91 for the general scale; 0.75 for “amnesia”; 0.76 for “absorption”; and 0.88 for the “depersonalization” scale (Zdankiewicz-Ścigała et al., 2020).

#### Analysis of the Results Obtained

A statistical analysis to test the hypotheses put forward was made in IBM SPSS Statistics, version 25. Key descriptive statistics were analyses with the use of the software, which made it possible to study the distributions of successive measured variables. Parametric tests were performed on all variables because skewness values did not exceed the conventional absolute value of 2. The hypotheses were tested with the use of a series of correlation analyses, regression analyses using a stepwise method of entering predictors into the model, and structural equation models (AMOS and GLS). The significance level was adopted at the classic threshold of α = 0.05.

#### Attachment Styles, Trauma, and Alexithymia and Dissociation in the Context of Alcohol Addiction

The main purpose of a multistage analysis was to verify the assumptions regarding the role of attachment styles, the intensity of early childhood trauma, and the level of alexithymia and dissociation in the development of addiction. Table 1 shows the demographic characteristics, descriptive statistics, and intergroup comparison.

**TABLE 1:** Demographic characteristics, attachment style, alexithymia, dissociation, and level of addiction in control group vs. alcohol addiction group.

	Control group				Alcohol addiction group				*F*	*p*	η^2^
	*M*	*SD*	95% CI^1^	95% CI^1^	*M*	*SD*	95% CI^1^	95% CI^1^			
Age	35.12	13.31	32.41	37.98	42.36	12.72	40.39	44.30	18.147	<0.001	0.067
**Attachment style**											
Secure attachment	44.75	9.01	42.70	46.69	37.55	10.19	35.87	39.26	27.380	<0.001	0.112
Anxious-ambivalent attachment	25.85	9.84	23.58	28.12	31.51	11.81	29.53	33.46	12.947	<0.001	0.057
Avoidant attachment	17.73	7.99	16.01	19.50	24.23	10.37	22.49	25.97	23.242	<0.001	0.096
**Alcohol addiction**											
Michigan Alcoholism Screening Test	0.82	1.13	0.60	1.05	33.93	17.04	31.25	36.60	338.373	<0.001	0.574
**Alexithymia**											
Difficulty in describing feelings	12.76	4.14	11.90	13.63	15.78	3.96	15.15	16.39	31.300	<0.001	0.117
Difficulty in identifying feelings	15.22	6.39	13.96	16.55	21.07	6.04	20.11	22.02	49.784	<0.001	0.175
Externally oriented thinking	17.72	4.19	16.88	18.61	19.25	4.75	18.46	20.07	6.102	<0.05	0.026
Alexithymia	45.82	12.53	43.23	48.46	55.54	11.86	53.53	57.55	33.619	<0.001	0.135
**Dissociation**											
Amnesia	10.73	2.84	10.18	11.36	13.62	4.55	12.94	14.37	28.121	<0.001	0.108
Absorption	24.11	7.95	22.47	25.82	28.89	9.84	27.33	30.46	14.618	<0.001	0.059
Depersonalization	8.26	3.03	7.70	8.92	9.74	3.75	9.17	10.34	9.736	<0.05	0.040
Dissociation	43.51	11.03	41.27	45.97	52.98	15.53	50.32	55.73	21.242	<0.001	0.096

*Mean (SD), ANOVA, η^2^, and (BCa) bootstrap confidence interval are listed. ^1^BCa, bootstrap confidence interval.*

In order to verify the hypothesis on the differences in attachment styles among subjects in the tested and control groups, a single-factor analysis of variance was carried out in the intergroup schema, which proved important effects for every attachment style: secure *F*(1,218) = 27.380; *p* < 0.001; η^2^ = 0.112; ambivalent *F*(1,216) = 12.947; *p* < 0.001; η^2^ = 0.057; avoidant *F*(1,218) = 23.242; *p* < 0.001; η^2^ = 0.096. Subjects addicted to alcohol showed significantly lower scores on the scale of secure attachment style and higher scores on the anxious-ambivalent and avoidant scales. In order to examine in detail the hypothesis on the intensity of traumatic experiences in the tested groups, a single-factor analysis of variance in the intergroup schema was also carried out, as a result of which it was proven that subjects addicted to alcohol showed significantly higher levels of general trauma intensity (*M* = 5.83; *SD* = 4.34) than subjects who were not addicted to alcohol (*M* = 3.17; *SD* = 3.02); *F*(1,224) = 24.585; *p* < 0.001; η^2^ = 0.099. Another stage of the analysis was the examination of hypotheses on the level of alexithymia and dissociation in tested groups. In order to check the significance of differences in the level of dissociation, a single-factor analysis of variance was also carried out, which proved a significant effect for the level of dissociation and its subdimensions: a general score *F*(1,199) = 21.242; *p* < 0.001; η^2^ = 0.096; absorption *F*(1,232) = 14.618; *p* < 0.001; η^2^ = 0.059; amnesia *F*(1,232) = 28.121; *p* < 0.001; η^2^ = 0.108; depersonalization *F*(1,232) = 9.736; *p* < 0.05; η^2^ = 0.040. Subjects addicted to alcohol showed significantly higher scores on all scales of dissociation. Also, a single-factor analysis of variance was carried out in the intergroup schema in order to determine the significance of differences between the tested groups, which proved a significant effect for this dimension and its subdimensions: a general score *F*(1,216) = 33.62; *p* < 0.001; η^2^ = 0.135; TAS-1 *F*(1,237) = 31.3; *p* < 0.001; η^2^ = 0.117; TAS-2 *F*(1,234) = 49.784; *p* < 0.001; η^2^ = 0.175; TAS-3 *F*(1,227) = 6.102; *p* < 0.05; η^2^ = 0.026. Before the commencement of more advanced statistical analyses, *r*-Pearson correlation analyses were carried out in order to determine whether the specific types of traumatic experiences relate to particular subdimensions of dissociation and alexithymia (see Tables 2, 3).

**TABLE 2:** Correlation matrix between traumatic experience and tendency to dissociation.

		Amnesia		Absorption		Depersonalization		Dissociation	
		C	A	C	A	C	A	C	A
Emotional neglect	*r*	0.111	**0.318**	0.198	**0.284**	0.068	**0.177**	0.218	**0.309**
	*p*	0.311	**0.000**	0.069	**0.001**	0.538	**0.033**	0.062	**0.000**
	*N*	85	**145**	85	**145**	85	**145**	74	**124**
Emotional abuse	*r*	0.089	0.089	0.172	0.124	0.124	0.079	0.187	0.061
	*p*	0.420	0.281	0.116	0.136	0.259	0.344	0.111	0.496
	*N*	85	147	85	147	85	147	74	125
Physical abuse	*r*	**0.288**	**0.179**	0.142	0.109	**0.301**	0.007	**0.275**	0.111
	*p*	**0.008**	**0.031**	0.196	0.191	**0.005**	0.937	**0.018**	0.217
	*N*	**85**	**146**	85	146	**85**	146	**74**	125
Threat to life	*r*	0.128	**0.312**	**0.243**	**0.253**	0.157	**0.194**	0.191	**0.275**
	*p*	0.245	**0.000**	**0.026**	**0.002**	0.154	**0.020**	0.105	**0.002**
	*N*	84	**144**	**84**	**144**	84	**144**	73	**122**
Sexual harassment	*r*	0.040	0.049	0.031	0.101	0.045	**0.197**	0.072	0.127
	*p*	0.716	0.558	0.783	0.230	0.681	**0.018**	0.544	0.160
	*N*	84	144	84	144	84	**144**	74	124
Sexual abuse	*r*	**0.399**	0.002	**0.253**	0.152	**0.450**	0.034	0.165	0.126
	*p*	**0.000**	0.985	**0.019**	0.070	**0.000**	0.686	0.159	0.168
	*N*	**85**	143	**85**	143	**85**	143	74	122
Trauma intensity	*r*	**0.312**	**0.299**	**0.298**	**0.271**	**0.345**	**0.217**	**0.332**	**0.325**
	*p*	**0.005**	**0.001**	**0.008**	**0.002**	**0.002**	**0.012**	**0.005**	**0.000**
	*N*	**79**	**132**	**79**	**132**	**79**	**132**	**71**	**112**

*C, control group; A, alcohol addiction group. Significant correlations are marked in bold. Source: Zdankiewicz-Ścigała (2017).*

**TABLE 3:** Correlation matrix between traumatic experience and alexithymia.

		Difficulty in describing feelings		Difficulty in identifying feelings		Externally oriented thinking		Alexithymia	
		C	A	C	A	C	A	C	A
Emotional neglect	*r*	**0.235**	**0.171**	**0.309**	**0.180**	−0.027	−0.012	**0.224**	**0.197**
	*p*	**0.028**	**0.039**	**0.003**	**0.031**	0.807	0.890	**0.037**	**0.026**
	*N*	**88**	**146**	**89**	**143**	87	138	**87**	**128**
Emotional abuse	*r*	**0.214**	0.097	**0.236**	0.146	−0.102	−0.114	0.154	0.035
	*p*	**0.046**	0.238	**0.026**	0.079	0.348	0.180	0.154	0.692
	*N*	**88**	149	**89**	145	87	140	87	129
Physical abuse	*r*	0.179	0.034	0.113	0.040	−0.151	**−0.189**	0.080	−0.066
	*p*	0.096	0.683	0.290	0.633	0.163	**0.026**	0.461	0.457
	*N*	88	147	89	144	87	**139**	87	128
Threat to life	*r*	0.145	0.083	0.165	**0.182**	0.006	0.104	0.133	0.122
	*p*	0.182	0.319	0.125	**0.031**	0.958	0.227	0.221	0.176
	*N*	87	145	88	**141**	86	136	86	125
Sexual harassment	*r*	0.059	−0.117	0.044	−0.066	−0.123	−0.104	−0.001	−0.129
	*p*	0.590	0.160	0.683	0.437	0.258	0.226	0.994	0.149
	*N*	87	146	88	143	86	138	86	127
Sexual abuse	*r*	0.064	−0.053	0.066	0.068	−0.027	−0.084	0.045	−0.028
	*p*	0.551	0.526	0.537	0.420	0.806	0.329	0.676	0.756
	*N*	88	145	89	142	87	137	87	126
Trauma intensity	*r*	**0.224**	0.081	**0.259**	0.129	−0.109	−0.160	0.164	0.055
	*p*	**0.043**	0.357	**0.018**	0.144	0.331	0.074	0.140	0.556
	*N*	**82**	132	**83**	129	82	126	82	117

*C, control group; A, alcohol addiction group. Significant correlations are marked in bold. Source: Zdankiewicz-Ścigała (2017).*

**TABLE 4:** Model matching values (1, reference and 2, after the removal of two paths).

	Secure		Avoidant		Anxious-ambivalent	
	attachment		attachment		attachment	
	Model 1	Model 2	Model 1	Model 2	Model 1	Model 2
χ^2^	2.26	7.30	0.23	5.49	2.04	6.60
χ^2^ (*p*)	0.132	0.063	0.631	0.139	0.154	0.086
CMIN/*df*	2.263	2.432	0.231	1.829	2.036	2.198
CFI	0.986	0.952	1.000	0.973	0.987	0.956
F0	0.005	0.016	0.000	0.009	0.004	0.013
90% LLCI (F0)	0.000	0.001	0.000	0.000	0.000	0.001
90% ULCI (F0)	0.037	0.061	0.016	0.050	0.035	0.057
GFI	0.997	0.989	1.000	0.992	0.997	0.990
RMSEA	0.069	0.073	0.000	0.056	0.062	0.067
CAIC	94.54	86.39	92.50	84.58	94.31	85.69
Hoetler	454	286	447	381	504	381

*CFI, comparative fit index; LLCI, lower level confidence interval; ULCI, upper level confidence interval; GFI, goodness of fit index; RMSEA, root mean square error of approximation; CAIC, consistent akaike information criterion.*

The strongest connection of dissociation, as a whole, and its particular dimensions relates to sexual abuse and emotional neglect. On the other hand, in a group of non-addicted, a significant correlation relates to a threat to life and physical abuse. From the perspective of an inclination to use the normative and pathological dissociation, it may be considered that non-addicted individuals present an inclination to normative dissociating, i.e., in situations compliant with a definition of trauma according to DSM-5 (APA, 2013). The analysis of correlations in the case of alexithymia indicates that important are the connections between emotional neglect, and additionally, emotional abuse in relation to alexithymia, as a whole, and dimensions related to difficulties in identifying and verbalizing emotions. It is important that such relations exist in the tested group, as well as in the control group.

#### Attachment Style, Trauma, Alexithymia, Dissociation, and Structural Equation Modeling

During the second stage, the more complex model of relations was tested with reference to theoretical assumptions on possible connections among multiple variables. As the biggest contribution to the understanding of the etiological path that fosters the development of addiction, we consider the inference below. In the assumed hypothetical model, variables were simultaneously taken into account, with reference to which research hypotheses were formulated, i.e., attachment styles, traumatic experiences in childhood, alexithymia and dissociation, and the level of alcohol addiction.

**FIGURE 2:**
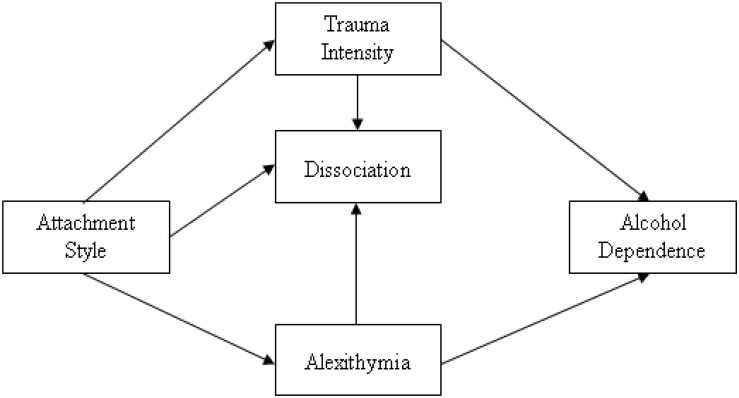
A final graphical model presenting the nature of dependencies between attachment styles and trauma, dissociation, and alexithymia, in explaining an inclination to alcohol addiction risk. Source: Zdankiewicz-Ścigała (2017).

In order to examine relations among variables, structural equation modeling was applied. With structural equation models (AMOS and GLS), the adjustment of a model was verified, which enabled explaining the occurrence of alcohol addiction. The model was tested for three attachment styles in parallel: secure, anxious-ambivalent, and avoidant. The first analyses confirmed the satisfactory adjustment of all models. In the majority of analyses in the model, two relations turned out to be statistically insignificant: between attachment styles and dissociation and between attachment styles and alcohol addiction. Due to this, a decision was undertaken to verify the adjustment of models after eliminating those paths (see Figure 2). The achieved solution provided models which were better adjusted to data. In order to check the stability, the obtained models were bootstrapped (1,000 repetitions, and additionally, Bollen–Stine’s correction was taken into account). Conclusions were based on the 95% value of the confidence intervals.

**FIGURE 3:**
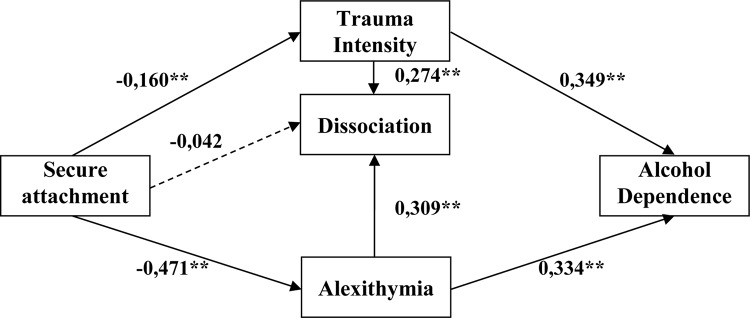
Standardized estimates for models taking into account the secure attachment style (dashed lines marked not significant paths). Source: Zdankiewicz-Ścigała (2017). *p < 0.05 and **p < 0.01.

In the case of a model for attachment style, Bollen–Stine’s correction confirmed its high stability (*p* = 0.063) (see Table 5 and Figure 3). Most paths in the model turned out to be statistically significant. As a result of conducted analysis, it was proven that the increase of alcohol addiction may be explained with the increase of an intensity of traumatic experiences and the intensity of alexithymia. A detailed critical ratios (CRs) analysis showed that the intensity of traumatic events is a significantly stronger predictor of addiction than alexithymia (CR = 4.29). It was also proven that the stronger secure the attachment style, the lower the intensity of traumatic experiences and the lower the level of an inclination to alexithymia. In addition, the achieved results enable us to make an assumption that an inclination to alexithymia influences the level of dissociation. The higher its level, the higher the probability of an inclination to pathological dissociation. The intensity of traumatic experiences is an important predictor of dissociation. Based on the obtained value of the CR rate, it is possible to state that the occurrence of alexithymia is a stronger predictor for the development of dissociation. In the model, according to theoretical assumptions, only the relation of secure style with dissociation and alexithymia turned out to be insignificant. The overall effect between a secure attachment style and dissociation was −0.231, and it may mostly be explained by an indirect impact of alexithymia and trauma intensity (−0.189). It is worth mentioning that a secure attachment style does not contribute to the development of alexithymia; however, deficits in the area of recognizing and understanding emotions may also emerge as a result of life-threatening surgeries or invasive traumas experienced in adulthood (e.g., long-term imprisonment and tortures). Affective disorders that emerged based on such traumas are considered as secondary alexithymia (Messina et al., 2014).

**TABLE 5:** Standardized estimates with 95% confidence intervals for models taking into account the secure attachment style.

Parameter			Estimate^1^	95%	95%	*p*^3^
				LLCI^2^	ULCI^2^	
Trauma intensity	←	Secure attachment	−0.160	−0.272	−0.041	0.003
Alexithymia	←	Secure attachment	−0.471	−0.558	−0.375	0.003
Dissociation	←	Secure attachment	−0.042	−0.157	0.075	0.458
Dissociation	←	Trauma intensity	0.274	0.131	0.393	0.004
Alcohol addiction	←	Trauma intensity	0.349	0.243	0.442	0.004
Alcohol addiction	←	Alexithymia	0.334	0.215	0.43	0.004
Dissociation	←	Alexithymia	0.309	0.199	0.421	0.002

*^1^Standardized estimate; ^2^Confidence intervals; ^3^*p*-value. Source: Zdankiewicz-Ścigała (2017).*

**FIGURE 4:**
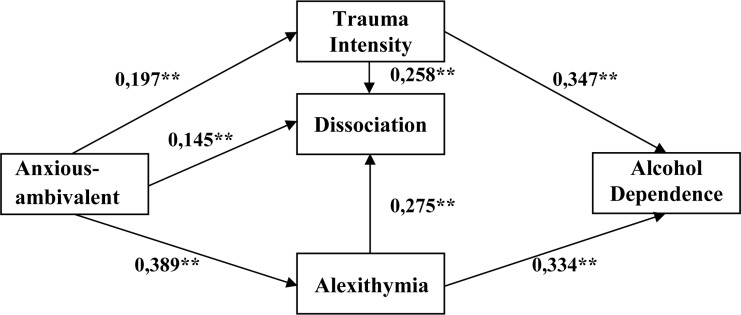
Standardized estimates for models taking into account the anxious-ambivalent attachment style. *p < 0.05 and **p < 0.01. Source: Zdankiewicz-Ścigała (2017).

Further analyses were carried out in reference to an anxious-ambivalent style (see Table 6 and Figure 4). In the case of a model for this style, Bollen–Stine’s correction confirmed a high stability of the tested model (*p* = 0.059). All paths tested in the model turned out to be statistically significant. It was found that the increase of alcohol addiction may be explained both by the increase in the strength of traumatic experiences and by the intensity of alexithymia. A detailed CR analysis showed that the intensity of traumas constitutes a significantly stronger predictor for the addiction than alexithymia (CR = 4.26). It was also proven that an anxious-ambivalent style relates to the increase in the strength of traumatic experiences, the intensity of alexithymia, and the increase of an inclination to dissociation. It was further proven in this model that an anxious-ambivalent attachment style, alexithymia, and trauma intensity significantly influence the level of dissociation. According to the CR value, the intensity of alexithymia and traumatic events are much more strongly related to the intensity of dissociation than an anxious-ambivalent attachment style. The overall effect between an anxious-ambivalent style and dissociation amounted to 0.302, and it may be half explained by an indirect impact of alexithymia and trauma intensity (0.158) and half explained by a direct impact of an anxious-ambivalent style. When the accuracy of a model with an anxious-ambivalent attachment style is tested, the presence of an earlier-described relation may be observed. An attachment style contributes to the development of alexithymia, and, to some extent, it relates to the exposure to traumas. Trauma and alexithymia, on the other hand, reinforce the tendency toward dissociation development. Important for the inclination to addiction are the level of alexithymia and the intensity of traumatic experiences. The stronger the trauma, the greater the affective dysregulation. Deficits in accurate processing of arousal activate stress and tension reflexively. The accessibility of alcohol and its fast effect in the form of tension release encourage individuals to refer to the substance.

**TABLE 6:** Standardized estimates with 95% confidence intervals for models taking into account the anxious-ambivalent attachment style.

Parameter			Estimate^1^	95%	95%	*p*^3^
				LLCI^2^	ULCI^2^	
Trauma intensity	←	Anxious attachment	0.197	0.07	0.302	0.004
Alexithymia	←	Anxious attachment	0.389	0.29	0.485	0.002
Dissociation	←	Anxious attachment	0.145	0.034	0.247	0.006
Dissociation	←	Trauma intensity	0.258	0.119	0.373	0.003
Alcohol addiction	←	Trauma intensity	0.347	0.242	0.439	0.004
Alcohol addiction	←	Alexithymia	0.334	0.216	0.43	0.004
Dissociation	←	Alexithymia	0.275	0.163	0.394	0.002

*^1^Standardized estimate; ^2^Confidence intervals; ^3^*p*-value. Source: Zdankiewicz-Ścigała (2017).*

**FIGURE 5:**
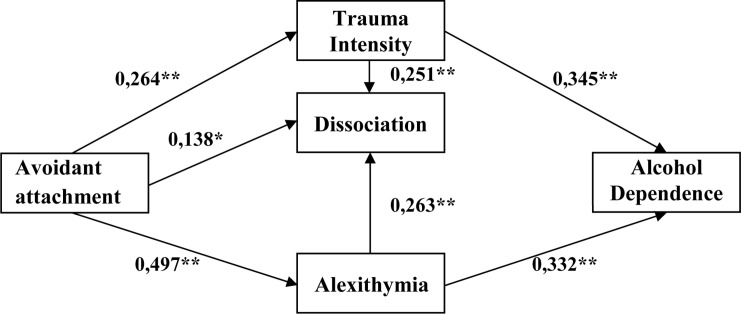
Standardized estimates for models taking into account the avoidant attachment style. *p < 0.05 and **p < 0.01. Source: Zdankiewicz-Ścigała (2017).

In the last tested model (see Table 7 and Figure 5), an avoidant attachment style constituted the main independent variable. In the case of a model for avoidant style, Bollen–Stine’s correction confirmed its high stability (*p* = 0.132). All paths tested in the model turned out to be statistically significant. It was found that the increase of alcohol addiction may be explained both by the increase in the strength of traumatic experiences and by the intensity of alexithymia. A detailed CR analysis showed that the intensity of traumatic events constitutes a significantly stronger predictor for the addiction than alexithymia (CR = 4.10). As a result of conducted analysis, it was also proven that the more avoidant the attachment style, the stronger the intensity of traumatic events and the higher the level of alexithymia and dissociation. Similarly, as in the case of an anxious-ambivalent style, the level of dissociation is conditioned by the avoidant attachment style, the level of alexithymia, and the intensity of traumatic experiences. According to the CR value, the intensity of alexithymia and the intensity of traumas are much stronger predictors for an inclination to dissociation than the impact of an avoidant attachment style itself. The overall effect between an avoidant attachment style and dissociation amounted to 0.335, and it may be explained, in the major part, by an indirect impact of alexithymia and trauma intensity (0.197) and, in the remaining part, by a direct impact of avoidant style (0.138). A detailed analysis of values among individually examined variables enables us to confirm the assumption verified previously that it is the avoidant attachment style that has the strongest negative impact on the development of a strategy for affect regulation and general emotional development. A relation between this attachment style and the development of alexithymia is extremely strong. From a psychological point of view, it is possible to state that emotional illiteracy, which emerged based on a very difficult attachment relation, increases the risk of psychopathology.

**TABLE 7:** Standardized estimates with 95% confidence intervals for models taking into account the avoidant attachment style.

Parameter			Estimate^1^	95%	95%	*p*^3^
			LLCI^2^	ULCI^2^		
Trauma intensity	←	Avoidant attachment	0.264	0.148	0.375	0.002
Alexithymia	←	Avoidant attachment	0.497	0.403	0.58	0.003
Dissociation	←	Avoidant attachment	0.138	0.015	0.267	0.024
Dissociation	←	Trauma intensity	0.251	0.109	0.374	0.003
Alcohol addiction	←	Trauma intensity	0.345	0.239	0.437	0.004
Alcohol addiction	←	Alexithymia	0.332	0.214	0.428	0.004
Dissociation	←	Alexithymia	0.263	0.154	0.371	0.002

*^1^Standardized estimate; ^2^Confidence intervals; ^3^*p*-value. Source: Zdankiewicz-Ścigała (2017).*

## Discussion

In the original model, authors assumed (see Figure 1) – in accordance with theoretical justification and the results of numerous studies – a direct impact of attachment styles on the inclination toward alcohol addiction. However, as a result of the path analysis, it turned out that the model is appropriately fitted to the data, but only when the impact path regarding the direct relationship between the attachment style and addiction was removed (see Figure 2). The verification of individual relationships was carried out for the three distinguished attachment styles separately. The purpose of this action was to explore the importance of individual attachment styles for the development of alexithymia and addiction in the context of trauma and dissociation. In the case of a model for the secure style, most of the paths in the model turned out to be statistically significant. Two factors turned out to be significant for the addiction itself: intensity of trauma and alexithymia. It is worth noting that the level of dissociation was affected by an inclination toward alexithymia. The relationship between a secure style and dissociation and alexithymia proved to be insignificant as expected. The obtained results should be considered as the verification of assumptions regarding the role of a secure attachment in the development of the ability to understand emotions and to regulate them. The more secure the attachment, the more developed the mechanisms of regulation and self-regulation. The essence of optimal development is, among others, the ability to mentalize (Allen et al., 2014), and the development takes place on the basis of the main caregiver’s emotional involvement. Important is the connection between the child and the caregiver with mutual responsiveness, which takes the form of emotional tuning of the parent and the child. Extremely important in this process are the “marked” emotional responses, which mean that the parent tunes into the child’s emotional state, expressing the same emotion, but supplemented with signals indicating that it is an act of reflection of the child’s condition, and not an expression of the parent’s pure emotions. The recurrence of such situations allows children (and later adults) to develop a sense of self and, in particular, the awareness of their own emotions. These parents’ “marked” emotional responses are fundamental for the development of the ability to regulate emotions in children and further in adults. Such individuals with developed competences regarding the processes of understanding and verbalization of emotions – as demonstrated in the model, the more secure the attachment, the lower the level of alexithymia (−0.471) – are characterized by the lack of alexithymia. In the case of this attachment, low-level trauma and dissociation were also noted. These are very important results, which confirm how important proper attachment is for affective development. However, it is worth adding that alexithymia itself has already been significantly associated with addiction (0.334), constituting a significant predictor of an inclination toward addiction.

Further, when the anxious-ambivalent style was introduced to the model, full verification of the adopted model was obtained. An anxious-ambivalent attachment favors the intensity of traumas, alexithymia, and thus an inclination toward addictions. Non-secure attachments, which favor experiencing strong negative emotions, rumination, and a sense of guilt with the absence of competences to understand and verbalize emotions, promote dysregulation, promote emotional tension (stress), and thus become a predictor of more frequent traumas and dissociation. Hence, another important examined path of dependency leads through trauma and alexithymia to dissociation and ultimately to addiction. In explaining the mechanisms of intoxication, Thorberg and Lyvers (2010) refer to the metaphor of a “safe base” that alcohol drinkers consider. According to them, alcohol intoxication is a substitute for a secure attachment that people who originated from anxious attachments did not have. Alcohol, in the case of individuals with a high level of alexithymia, may constitute a source of relaxation and pleasant comfort, allowing them, also from this “safe base,” to experience and show emotions. The illusion of this “safe shelter” also relates with emotional unrest and the lack of control and self-control, which often results in impulsive and risky behaviors. Cognitive distortions connected with the understanding of alcohol drinking, as demonstrated in Dragan’s (2016) studies, aggravate addiction on the one hand and deficits in understanding and regulating emotions, i.e., the level of alexithymia, on the other.

The mechanism described is even more visible in dependencies obtained during the verification of the third model, where an avoidant attachment style has been introduced. As shown, dependency paths have proven to be very significant between the analyzed variables. The increase in alcohol dependence may be explained by an avoidant attachment through the intensity of traumatic experiences on the one hand and the severity of alexithymia on the other. The relation between the avoidant attachment and alexithymia (0.497) is definitely more important compared with the anxious-ambivalent one (0.398). Similar relationships related to the prediction of alexithymia based on attachment styles were obtained by Lyvers et al. (2019). It is worth mentioning that the following are distinctive for the avoidant attachment in the development of emotional regulation patterns: (1) blocking emotions and limited access to sadness and anxiety, (2) the absence of emotional content integration in the process of information processing and action planning, and (3) a distance from threatening thoughts, emotions, and experiences (Marszał, 2014). In fact, these are indicators of developing alexithymia. Since the origins of alexithymia are tracked in anxious attachment styles, these deficits may be considered a very important factor, not only strengthening the distortions in the reception of emetogenic information but also contributing to decompensation at the neurophysiological level. The more anxious the attachment, the stronger the disorder and the greater the risk of exposure to trauma, both relational traumas (including traumas of betrayal with a small “t”) and traumas with a capital “T.” As indicated by Schore (2009b), as a result of chronic stress in which a child is brought up, inefficient regulation of the autonomic nervous system (ANS) by higher centers in the central nervous system (CNS) takes place, which is manifested as a disorder of the central regulation of the sympathetic nervous system and the hypothalamus–pituitary–adrenal axis. “The loss of such regulation means that in a situation of stress, the mode of associated mutual autonomous control gives way to the mode of associated non-mutual autonomous control, as a result of which an extremely high state of arousal arises of both sympathetic and parasympathetic systems” (Schore, 2009b, p. 94). Particular disorders may take the form of individualized symptoms with usually underlying traumatic development. Both alexithymia and dissociation become independent factors which increase the risk of post-traumatic disorders’ development, i.e., post-traumatic stress disorder (PTSD) and complex PTSD (cPTSD), or other mental disorders in a child and further—in the course of development – in an adult. The connection between alexithymia and PTSD is indicated, among others, in a meta-analysis carried out by Frewen et al. (2008). Dissociation is considered a feature present in post-traumatic disorders (APA, 2013). Worth remembering are the mutual relationships between alexithymia and dissociation in the etiopathogenesis of PTSD-like disorders, where in numerous cases addiction is also present, as evidenced by the results of multiple studies, also carried out in Poland, and a dual diagnosis is not uncommon (e.g., Dragan, 2016).

To sum up, the study was the first to analyze the mechanisms underlying alexithymia in the context of trauma and dissociation in the group of alcohol addicts based on the attachment paradigm. The fact that three attachment styles have been taken into account separately indicates clearly the origin of alexithymia as a consequence of disorders in early childhood attachment and developmental traumas. Alexithymia appears as a variable which connects non-secure attachment with trauma, dissociation, and addiction. However, in the context of the presented analyses, it is worth taking into account that its role in regulating emotional tension and affective overwhelming may be twofold. On the one hand, it serves to suppress anxiety and concern and, in general, to deal with stress or negative affect (anxious-ambivalent attachment) and, on the other hand, to disinhibit and reveal emotions (avoidant attachment). In both types of attachments, disorders in the development of emotional regulation processes are slightly different, and the characteristics of alexithymia also differ (Bermond, 1997). The regulatory role of alexithymia in coping with stress and negative affect is indicated in the results of the study by Lyvers et al. (2018a), as well as its role in the intensification of emotional sensations under the influence of alcohol (Thorberg et al., 2016). The context in which the deficits described under the concept of alexithymia develop determines the form of the disorder and defines which dominating emotions become disturbed and distorted.

## Conclusion

In the light of gathered and quoted data, it may be considered that the proposed theoretical model turned out to be useful to deepen the role of understanding individual analyzed factors in the development of disorders of emotional processes related to alcohol dependence development. Our studies revealed how important it is to investigate the role of individual variables in the context of development. An extremely important element of the scientific achievement presented here is showing pillars of traumatic development, i.e., alexithymia and dissociation in their cumulative impact, on the development of emotional disorders resulting in addiction. Another crucial issue is also analyzing their pathogenic impact on building romantic relationships in adulthood (Thorberg and Lyvers, 2006; Zdankiewicz-Ścigała and Ścigała, 2018b). The absence of empathy, impulsiveness, non-adaptive strategies of emotion regulation, suppression of negative emotions, and depression (Martinotti et al., 2009; Lyvers et al., 2018b) all contribute to experiencing a strong fear of intimacy and rejection in a relationship, ending in a persistent addiction, despite attempts to overcome it.

## Future Directions

The results, which authors have obtained, are undoubtedly valuable since they show the role of alexithymia as a key factor combining attachment styles, trauma, and dissociation in the pathomechanism of addiction development. In subsequent studies, it is worth verifying the model by examining young individuals addicted to alcohol, controlling their temperament, the type of dominant negative emotions, and subjects’ sex. The purpose of such actions would be to verify two paths of addiction development: (1) as a consequence of anxiety, stress, and emotional tension regulation and (2) as a trigger factor of emotions that motivate display of risky behaviors.

## Data Availability Statement

The datasets generated for this study are available on request to the corresponding author.

## Ethics Statement

All procedures performed in studies involving human participants were in accordance with the ethical standards of the Institutional and/or National Research Committee and with the 1964 Declaration of Helsinki and its later amendments or comparable ethical standards. The study was reviewed and approved by the SWPS University of Social Sciences and Humanities Ethics Committee.

## Author Contributions

EZ-Ś made substantial contributions to the conception, design of the work, and acquisition. DŚ analyzed the data. EZ-Ś and DŚ interpreted the data for the work, drafted the work or revised it critically for important intellectual content, approved the final version to be published, and agreed to be accountable for all aspects of the work in ensuring that questions related to the accuracy or integrity of any part of the work are appropriately investigated and resolved.

## Conflict of Interest

The authors declare that the research was conducted in the absence of any commercial or financial relationships that could be construed as a potential conflict of interest.
